# Uncovering the early and conserved molecular mechanisms of root nitrogen foraging in model and crops

**DOI:** 10.1186/s12864-026-12736-5

**Published:** 2026-03-10

**Authors:** Ying Li, Ryan M. Patrick, Charles Peacock, Leo Koenigsfeld, Tara M Rock, Eleonore Bouguyon, Emily Kuhn, MarySara Albert, Kranthi Varala, W. Richard McCombie, Chia-Yi Cheng, Sandrine Ruffel, Gloria Coruzzi

**Affiliations:** 1 Center for Genomics and Systems Biology, Department of Biology, New York University, New York, NY 10003 USA; 2 Department of Horticulture and Land Architecture, Purdue University, West Lafayette, IN 47907 USA; 3 Center for Plant Biology, Purdue University, West Lafayette, IN USA; 4 School of Biological Sciences, Illinois State University, Normal, IL USA; 5 Cold Spring Harbor Labs, Cold Spring Harbor, NY USA; 6 Department of Life Science, National Taiwan University, Taipei, Taiwan; 7 Institute for Plant Sciences of Montpellier, Univ. Montpellier, INRAE, CNRS, Montpellier, France

**Keywords:** Nitrogen, Root foraging, Comparative transcriptomics, Gene networks, Split-root, Systemic signaling

## Abstract

**Background:**

Nitrogen (N) foraging, the ability of plants to promote preferential root growth in N-rich patches of soil, is fundamental to the competitiveness and wellbeing of plants. A unique “split-root” system, where a heterogenous N environment stimulates root foraging, provides a powerful experimental model to study the mechanisms underlying root foraging in model (Arabidopsis) and/or crop plants.

**Results:**

We used the split-root set up to capture early molecular events involved in systemic N-signaling after exposure to a heterogeneous N signal, through time-course transcriptomic analysis across shoots and roots of Arabidopsis. We found that a histone methyltransferase, SET DOMAIN GROUP 8 (SDG8), is necessary for root N-foraging, suggesting a previously unknown role for chromatin regulation in mediating the preferential root growth response to colonize N-rich patches. To determine if the underlying molecular mechanism is conserved in evolution, we compared the root foraging behavior from model-to-crop (Arabidopsis, tomato and maize). Our analysis showed the model and crop species shared a root N-foraging growth response, with some variation among specific genotypes. Interestingly, we observed both shared and distinct transcriptional responses to heterogenous N environments among these three species.

**Conclusions:**

Our study has generated insights into the molecular basis of root N-foraging, with the potential to improve nutrient use efficiency in crop plants in a heterogeneous field environment.

**Supplementary Information:**

The online version contains supplementary material available at 10.1186/s12864-026-12736-5.

## Background

Nitrogen (N) is a macronutrient essential for plant growth [1]. In temperate and well-aerated soil, nitrate (NO_3_^−^) is the major form of N available for plants, and acts as a signal to trigger genome-wide transcriptional reprogramming to facilitate N uptake and assimilation [1]. The abundance of nitrate in the soil often varies by orders of magnitude in space and time [2]. To optimize nitrate uptake, plants have evolved remarkable plasticity of enhancing root growth specifically in the N-rich soil patches —a phenomenon known as “root foraging” [2, 3]. Understanding the molecular and regulatory mechanism of root foraging for N is critical to improving nutrient uptake efficiency of plants, a trait with great relevance to agricultural productivity and environmental sustainability.

**Fig. 1:**
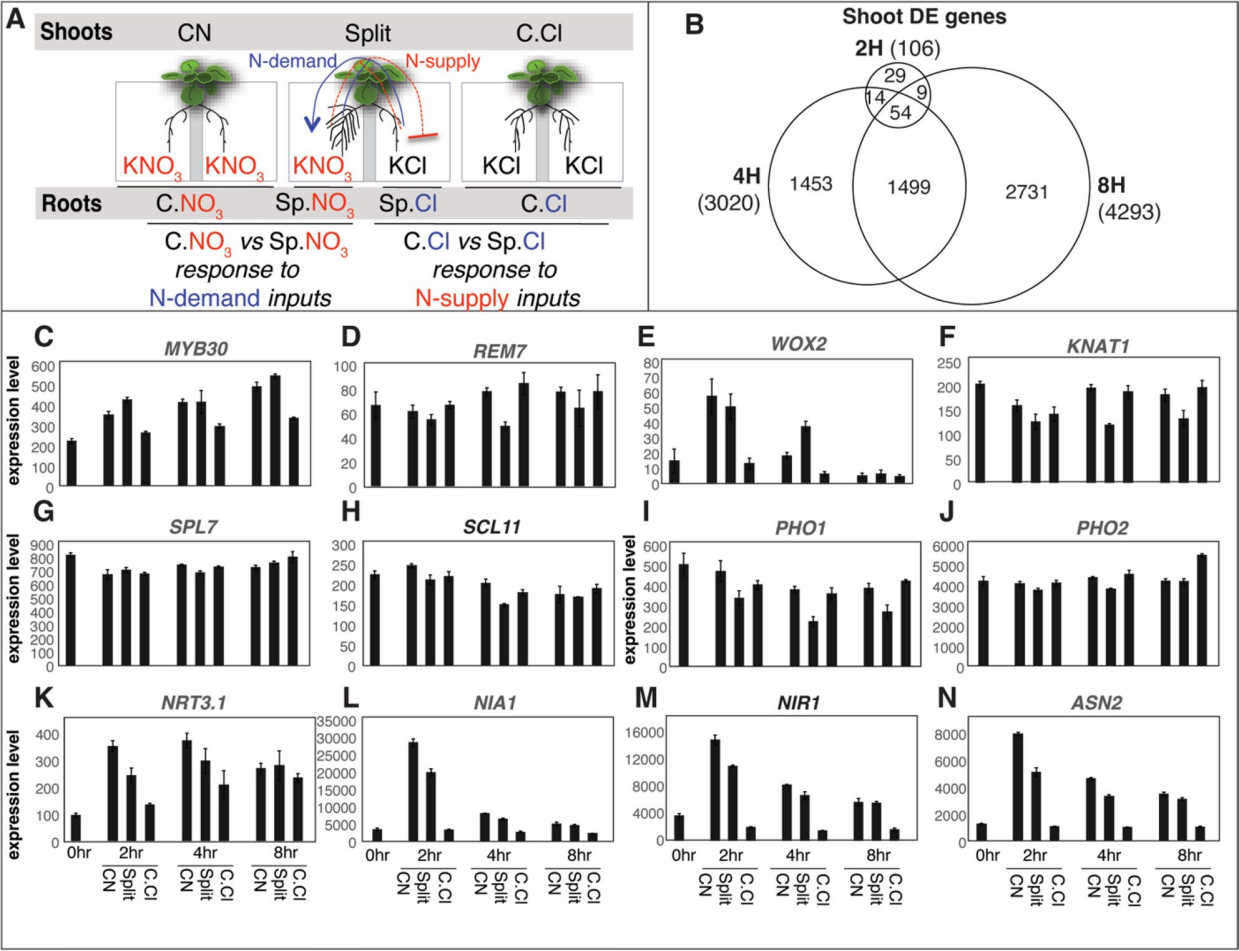
Heterogeneous nitrogen treatments triggered widespread transcriptional responses in the shoots in Arabidopsis. **A** Schematic representation of the split-root experimental setup studying the root foraging mechanism of Arabidopsis. Roots of a single plant are split into two halves, and each exposed to distinct N-level environments: either N-supply (Sp.NO_3_), or N-deplete (Sp.Cl). In parallel, roots of control plants are also “split” and exposed to homogeneous N-supply (C.NO_3_) or homogeneous N-deplete conditions (C.Cl). **B** Venn diagram showing the overlap of differentially expressed genes in the shoots at 2, 4, and 8 h after the onset of heterogeneous nitrogen (N) treatment. The differentially expressed genes were determining using one-way ANOVA model with the factor “treatment” at three levels (C.NO_3_, split and C.Cl) in R, with cutoff of False Discovery Rate of 0.1. **C**–**N** Expression profiles of selected genes in the shoots under C.NO₃, split (heterogeneous), and C.Cl conditions at 0, 2, 4, and 8 h as measured by RNA-seq. Genes encoding transcription factors (**C**–**H**) and phosphate transporter PHO1 and its regulator PHO2 (**I**, **J**) exhibited heterogeneous-N-specific expression patterns. In contrast, several nitrogen metabolism related genes, including nitrate transporter (**K**) and key enzymes (**L**–**N**), displayed expression patterns corresponding to the total nitrogen availability. The mean of normalized expression levels across three biological replicates are plotted, and the error bars represent standard error of the mean

To investigate the underlying mechanisms of root foraging, a unique “split-root” experimental system was developed during the late 19th century to study root growth responses to uneven distribution of essential minerals in the environment [4, 5]. In the N split-root system, roots of a *single plant* are split into two halves, and each exposed to distinct N-level environments: either N-supply (Sp.NO_3_), or N-deplete (Sp.Cl) [3] (Fig. 1A). The heterogeneous N environment (i.e. the “Sp.NO_3_+Sp.Cl” treatment) triggers root foraging, resulting in an enhanced root growth in the Sp.NO_3_ compartment compared to the root of the same plant in the Sp.Cl compartment. In parallel, roots of control plants are also “split” and exposed to *homogeneous* N-supply (C.NO_3_) or *homogeneous* N-deplete conditions (C.Cl) (Fig. 1A). Importantly, the growth of the Sp.NO_3_ roots is greater than that of the control C.NO_3_ roots, even though they are exposed to the same local [NO_3_^−^], indicating that the enhanced Sp.NO_3_ root growth is triggered by a *systemic* “N-demand” signal indicating the lack of NO_3_^−^ in part of the soil environment (represented by the blue lines in Fig. 1A). Similarly, the growth of the Sp.Cl roots is less than that of the C.Cl roots, indicating that the inhibited root growth was triggered by a *systemic* “N-supply” signal represented by red lines in Fig. 1A. Overall, the response to systemic N signals is observed as a robust difference, in root growth phenotype or gene expression, between heterogeneous N conditions and the corresponding homogeneous N conditions (i.e. C.NO_3_ roots vs. Sp.NO_3_ roots, or Sp.Cl roots vs. C.Cl roots) (Fig. 1A) [3]. Therefore, the split-root system enables investigators to distinguish root responses to systemic N signals from the responses to local N availability – which are undistinguishable in standard homogeneous N treatments [6]. The split-root system has greatly facilitated the mechanistic investigation of N root foraging, especially in the molecular genomic era [3, 6, 7]. Interestingly, the systemic N signaling also requires shoots, as plants without the shoots lose the ability to respond to systemic N signals while retaining responsiveness to local N signals [3]. It is reasonable to speculate that N root foraging involves multiple molecular players working in concert between shoots or roots, coordinated by long-distance signaling molecules traveling between the two organs. While molecular inquiry started with a focus on roots [3, 7], later studies has shifted to root-shoot signaling. Indeed, long-distance signaling molecules have been uncovered to mediate root foraging for N, including hormone cytokinin [3, 8, 9] and small peptides C*-*TERMINALLY ENCODED PEPTIDE (CEP) and CEP DOWNSTREAM (CEPD) [10, 11].

Several questions remain unanswered: *first*, what shoot–root communications are involved in the early N-signaling events in the N foraging? It has been reported that N-signaling occurs as early as in minutes and hours after the exposure to N changes [12]. Time course transcriptomic profiling at early time points in both shoots and roots, using a split-root setup, was needed to complement a previous study that focused on shoot transcriptome profiling at single time point (24 h) [8]. *Second*, compared to transcription factors (TFs), the role of chromatin regulation in mediating response to heterogenous N-supply remains unclear. It was previously reported that TFs, such as TEOSINTE-BRANCHED1,-CYCLOIDEA,-PCF-DOMAIN-FAMILY-PROTEIN20 (TCP20), are important for directing root foraging [7]. TFs regulate the transcription activity of target genes in the context of local chromatin landscape, which are controlled by various chromatin modifications [13]. However, the knowledge on the specific role of chromatin modifying proteins in regulating heterogenous N responses is lacking. The chromatin factor HIGH NITROGEN INSENSITIVE 9 (HNI9) was shown to regulate gene expression in response to a high N supply [14, 15]. Moreover, an Arabidopsis histone methyltransferase, SET Domain-containing Group 8 (SDG8), was shown to be necessary for the classic root response to homogeneous N changes [16], but its relevance to systemic N signaling in the heterogenous N environments has not been investigated. *Finally*, an essential and intriguing question is whether conserved molecular mechanisms are employed across different plant species that exhibit N-triggered root foraging phenotype, such as Arabidopsis [3] and maize [17]. A cross-species transcriptomic comparison, particularly one involving studies with comparable experimental setup and sampled time points, could illuminate which aspects and components of root plasticity behavior are conserved and which are species-specific. For example, one interesting question is whether a wild species evolved in the natural environment with limited N (e.g. Arabidopsis) and a crop species that has been through breeding and selection in fertilized soil (e.g. maize or tomato) share similar root foraging responses and underlying molecular circuits.

To fill these knowledge gaps, we conducted a comprehensive transcriptomic analysis using the split-root system. We assayed multiple early time points, from time 0 to 8 h, from Arabidopsis shoots and roots separately, to investigate early root-shoot-root signaling relay that is essential to root foraging. We uncovered that SDG8, the histone methyltransferase, is required for root N foraging. Finally, by comparing transcriptomic responses to heterogenous N conditions across Arabidopsis, tomato, and maize, we identified both shared and unique molecular circuits involved in the N foraging response across different plant species.

## Methods

### Arabidopsis split-root treatment and RNA-Sequencing

Split-root experiments were performed as previously described in [3] (Fig. 1A) using *Arabidopsis thaliana* (Col-0) seeds obtained from the Arabidopsis Biological Resource Center (ABRC stock No. CS70000). A root foraging phenotype was observed after 4 days, consistent with previously reported in [3]. Shoots and roots samples were harvested at 0, 2, 4, and 8 h after exposure to the heterogeneous N-treatments or control homogeneous N-treatments. For each time point and each N condition, 3 biological replicates were collected, each consisting of pooled tissues from 9 to 10 seedlings. Total RNAs were extracted from the tissues using the mirVana™ miRNA isolation kit (ThermoFisher Scientific) following the total RNA extraction protocol. Next, 5 µg total RNA of each sample was used to prepare RNA-Seq libraries following an in-house protocol using NEXTflex DNA Barcodes (Bioo Scientific). Then, 12 RNA-seq libraries were pooled for sequencing in 1 lane of Illumina Hi-Seq platform with the paired-end 100 bp format (in collaboration with W.R. McCombie at the Cold Spring Harbor Laboratory). For the phenotypic comparison of *sdg8* and WT, the *sdg8-5* seeds were previously described in [16] and the phenotypes were compared 4 days after the split-root treatment as described in [3].

### Arabidopsis RNA-Seq data analysis

Approximately 32 million reads were generated for each RNA-Seq library. The raw sequencing reads were trimmed with an in-house Perl script to remove adaptors and low quality reads, and then mapped to Arabidopsis TAIR10 genome using Bowtie2 [18]. Across the libraries, 73–83% of the reads mapped to genome, indicating satisfactory RNA-Seq library quality. The genome-mapped reads were then used to calculate the gene expression level using HTSeq [19] based on the TAIR10 genome annotation. The genome-wide expression matrix was then normalized to minimize between-lane variation using Full-Quantile normalization method [20] in the EDAseq package [21], for shoots and roots separately.

*Shoot transcriptome analysis*: the shoot transcriptome at every time point was analyzed separately (2 h, 4 h and 8 h). For each time point, low expressed genes (max expression level < 2^5^) were removed. The remaining genes (approximately 18,400 genes, 66% of the whole gene space) were then analyzed using a one-way ANOVA model with the factor “treatment” at three levels (C.NO_3_, split and C.Cl) in R. The *null hypothesis* of the one-way ANOVA is that the expression levels of *gene x* under all three conditions are the same. Any gene with expression significantly differing from this null hypothesis (at a False Discovery Rate [22] of 10%) was identified as a differentially expressed gene (DEG). The specific expression patterns of DEGs were further grouped based on a *post-hoc* Tukey’s test [23] in R. The Tukey’s test performed the following three pairwise comparisons: (i) C.NO_3_ vs. C.Cl, (ii) split vs. C.Cl, and (iii) split vs. C.NO_3_, with a cutoff of p-value < 0.05. The output of the three pairwise comparisons can be presented by a three-digit Tukey code T_1_T_2_T_3_ (T_n_ = 1, 0, or -1). T_1_ represents the result of C.NO_3_ vs. C.Cl, as follows: T_1_ = 1: C.NO_3_ > C.Cl; T_1_= -1: C.NO_3_ < C.Cl; T_1_= 0: C.NO_3_ is not significantly different from C.Cl. Similarly, T_2_ represents the result of split vs. C.Cl and T_3_ represents the result of split vs. C.NO_3_. Based on the Tukey code, the DEGs were categorized into eight (for 2 h) or 17 (for 4 h and 8 h) different regulation groups. For example, DEGs that are determined to be (i) C.NO_3_ *>* C.Cl, (ii) Split *>* C.Cl, and (iii) Split is not significantly different from C.NO_3_, form a regulation group. Finally, all eight regulation groups from the 2 h dataset, and 11 out of the 17 regulation groups from the 4 h and 8 h were discussed in the results, because these groups have clearly defined pattern (i.e. at least two out of the three pairwise comparisons are significant). These 11 groups include 75%–78% of the DEGs.

*Root transcriptome analysis*: we first developed a statistical test in R (www.r-project.org) to determine the level of dependency between the Sp.NO_3_ and Sp.Cl root transcriptome data, since they are harvested from the same plants. The result suggested that the Sp.NO_3_ and Sp.Cl root transcriptomes are interdependent, which is not surprising given that they are from the same plants. Due to the interdependency of Sp.NO_3_ and Sp.Cl root transcriptomes, pairwise comparisons are more suitable than a differential method integrating all four conditions together (C.NO_3_, Sp.NO_3,_ Sp.Cl, and C.Cl). Therefore, we developed a data analysis pipeline consisting of Perl scripts (www.perl.com) and R scripts (www.r-project.org) to automatically process the root transcriptome matrix to identify DEGs with various regulation patterns, using a pairwise rank product (RP) method [24]. In detail, the root transcriptome matrix was first split by time point. For each time point, the transcriptome data was log transformed and low expression genes were filtered out (max log 2 expression level < 5). Next, RP [24] was used to perform the following six pairwise comparisons: (i) C.NO_3_ vs. Sp.NO_3_, (ii) C.NO_3_ vs. Sp.Cl, (iii) C.NO_3_ vs. C.Cl, (iv) Sp.NO_3_ vs. Sp.Cl, (v) Sp.NO3 vs. C.Cl, and (vi) Sp.Cl vs. C.Cl, with a cutoff of FDR ≤ 10% at each time point. For each gene, the output of the six pairwise comparisons can be presented by a six-digit RP code R_1_R_2_R_3_R_4_R_5_R_6_ (R_n_ = 1, 0, or -1). For example, R_1_ represents the result of C.NO_3_ vs. Sp.NO_3_, as follows: R_1_ = 1: C.NO_3_ < Sp.NO_3_; R_1_= -1: C.NO_3_ > Sp.NO_3_; R_1_= 0: C.NO_3_ is not significantly different from Sp.NO_3_. Similarly, R_2_ to R_6_ represent the results of (ii) C.NO_3_ vs. Sp.Cl, (iii) C.NO_3_ vs. C.Cl, (iv) Sp.NO_3_ vs. Sp.Cl, (v) Sp.NO3 vs. C.Cl, and (vi) Sp.Cl vs. C.Cl. Based on the RP code, the root DEGs were categorized into 59, 73 and 82 regulation groups, for 2 h, 4 h, and 8 h transcriptomes separately (Additional File 2 Table S3).

### Arabidopsis shoot–root correlation analysis

To identify inter-organ correlated gene pairs, the normalized transcriptome of shoots and roots from the same plants over time points were used as input datasets for correlation analysis. For example, inter-organ correlated gene pairs for C.NO_3_ plants were calculated from C.NO_3_ shoots and C.NO_3_ roots (Fig. 1A); inter-organ correlated gene pairs for C.Cl plants were calculated from C.Cl shoots and C.Cl roots. For split-root plants, since the Sp.NO_3_ roots and Sp.Cl roots share the same shoots, the inter-organ correlated gene pairs for the N-replete side was calculated from split-root shoots and Sp.NO_3_ roots, while the inter-organ correlated gene pairs for the N-deplete side was calculated from split-root shoots and Sp.Cl roots. In each correlation analysis, the input transcriptome data were filtered based on low expression value cutoff (max log2 expression level > 5), and a dynamic range (max expression level / min expression level) > = 2, to avoid calculating correlation on unchanged genes (which will give a high correlation coefficient but not informative since the genes are constant between samples). After filtering, correlation coefficients and p-values were calculated between each gene in the shoot transcriptome and each gene in the root transcriptome over time using the *rcorr* function from the Hmisc R package. The p-values were then corrected for multi-testing error using the p.adjust function (FDR method) in R. An FDR cutoff of 0.001 was selected to be an ideal cutoff based on an FDR sensitivity test. This FDR cutoff is equivalent to (and slightly more stringent than) a correlation coefficient cutoff of |r|>0.9. Next, the significantly correlated shoot–root gene pairs were filtered based on whether the shoot gene and the root gene in the gene pairs are also identified as differentially expressed in the shoots and in the roots, separately, across different N treatments at any time point. As a result, 10,783, 6231, 7750, and 8365 gene pairs were identified for C.NO_3_, Sp.NO_3_, Sp.Cl and C.Cl, separately. Later, the identified gene pairs were compared with experimentally-determined trafficking RNAs reported previously [25], to select gene pairs where at least one transcript was reported to travel between shoots and roots. GO term analysis was performed with the Biomaps function in Virtual Plant [26] and AgriGO [27]. The resulted network was visualized in Cytoscape [28], and the functional enrichment analysis of the network was performed by BINGO using the BINGO plugin in Cytoscape [29].

### Tomato split-root treatment

Tomato seeds (Heinz 1706 and M82) were sterilized in 20% household bleach (5% sodium hypochlorite) for 30 min followed by rinsing six times with sterile water. Next, seeds were germinated on moist sterile filter paper in foil wrapped petri dishes in a growth chamber at 28 °C for 5 days. After germination, the tomato seedlings were mounted in foam plugs situated in custom-made black foam-board lids, allowing the roots to be immerse in 900 mL (+ N) growth medium (0.2 mM KH_2_PO_4_, 0.2 mM MgSO_4_, 0.05 mM KCl, 12.5 µM H_3_BO_3_, 1 µM MnSO_4_, 1 µM ZnSO_4_, 0.5 µM CuSO_4_, 0.1 µM H_2_MoO_4_, 0.1 µM NiSO_4_, 10 µM Fe-EDDHA) with 1.2 mM KNO_3_ and 0.8 mM Ca(NO_3_)_2_, in a plastic 1 L container. Air pumps were used to aerate the growth medium. The seedlings were placed in the growth chamber and grown for 7 days under a 16 h:8 h day/night cycle at 24 °C/20°C and 100 µmol/s^− 1^/ m^− 2^ white light. The growth media was refreshed every 3 days. After growing for 7 days in the growth chamber, each homogenous hydroponic system was moved to the greenhouse and grown for 7 more days under a 14 h/10 h day/night cycle at 24 °C/18°C, with approximately 500–700 µmol/s^− 1^/ m^− 2^ of sunlight supplemented with artificial light. Growth media was refreshed every 3 days. After 7 days of growth in the greenhouse, root cutting was performed to trim the root systems to just two lateral roots of equal length. Following root cutting, aeration for the plants was paused for 2 days to allow healing of the wounds. After a 4 d recovery period following root cutting, plants were grown for 2 days in starvation media (i.e. -N medium, identical to + N media except the 1.2 mM KNO_3_ and 0.8 mM Ca(NO_3_)_2_ were replaced by 1.2 mM KCl and 0.8 mM CaCl_2_). Next, the two roots were placed (“split”) into two separate containers in a homogenous or heterogonous hydroponic growth system, while the plant stalk was supported by wooden skewer. The containers were filled with solution for either (i) + N treatment or (ii) -N treatment. For each genotype, 6 plants were treated with C.NO_3_ system (N+/N+), 6 plants in a C.Cl system (N-/N-), and 12 plants in a Sp.NO_3_ /Sp.Cl system (N+/N-). After 7 days of growth in the heterogeneous or homogenous “split” hydroponic system, plants were then removed for root phenotyping using the Gia Roots software [30]. Moreover, roots were dried at 60 ˚C for 1 week before weighing to measure dry biomass.

### Tomato split-root transcriptome profiling and analysis

Tomato plants (Heinz 1706 and M82) were grown in split-root experiments as described above with a few minor modifications: seeds were sterilized and germinated on half-strength MS agar plates for one week in the growth chamber, and then seedlings were transferred to the hydroponic systems and grown for additional 5 days; next, they were transferred to the greenhouse conditions and grown for 5 days, before root cutting and heterogeneous/homogenous N-treatments as described above. Specifically, after 2 days of starvation media treatment, the plants were transferred to C.NO_3_, C.Cl, or Sp.NO_3_/Sp.Cl systems for 6 h. Root and shoot tissue samples were collected, combining three individuals per biological replicate, flash frozen in liquid nitrogen, then ground to powder. RNA was extracted from ground tissue samples with Plant RNeasy Kit (Qiagen) using RLC buffer for shoot samples and RLT buffer for root samples. Poly(A) enrichment, library construction, and sequencing (Illumina NovaSeq 6000 platform, paired end, 2 × 150 bp) were performed by the Purdue Genome Sequencing Core, with three replicates for root samples and four replicates for shoot samples in each genotype and treatment condition. Reads were trimmed with cutadapt and aligned to the *S. lycopersicum* v3.0 genome [31] using TopHat2 [32]. Gene counts for ITAG v3.2 annotation features were determined using HTSeq (19). Differentially expressed genes in roots and shoots in response to systemic N signals were determined from the interaction between genotype and condition using DESeq2 [33] with an FDR < 0.05.

### Maize split-root plant growth and treatment

Maize seeds (B73) were first surface sterilized with 10% bleach and 0.1% Tween 20, then imbibed in saturated CaSO_4_ solution for 5 h. The seeds were then allowed to germinate at 28 ˚C in the dark for 4 days, between paper towers pre-soaked with saturated CaSO_4_ solution. The plants were then transferred to a growth room with a 14 h:10 h day/night cycle. The day temperature was 28 ˚C, with a light intensity of 270–310 µM s^− 1^ m^− 2^ at the canopy level, while night temperature was 22 ˚C. Germinated seedlings were first grown in black sand with water for 7 days. Plants with 2 to 3 leaves were then transferred to hydroponic systems with nutrient solution as described in [34], while N was supplied as 0.25 mM Ca(NO_3_)_2_. Hydroponic systems were aerated every day for 4 h in the afternoon, unless specified. During this period, the medium was refreshed every 3 days. After 9 days in the hydroponic system, the embryonic roots (primary roots and seminal roots) were removed, and the crown roots were trimmed down to two equal-length crown roots (at least ~ 5 cm long); aeration was skipped on the day of cutting. The plants were then allowed to recover for 2 days in the same nutrient solution. Next, the seedlings were transferred to starvation solution with Ca(NO_3_)_2_ removed from the nutrient solution, for 2 days. Maize plants were then treated in the “split-root” condition, as follows: (1) in heterogeneous condition, a seedling was mounted on top of two adjacent containers with one root in nutrient solution supplied with 2 mM Ca(NO_3_)_2_ (Sp.NO_3_) and the other root in nutrient solution supplied with 2 mM CaCl_2_ (Sp.Cl); (2) in homogeneous condition, both roots were in nutrient solution supplied with 2 mM Ca(NO_3_)_2_ (C.NO_3_), or both roots were in nutrient solution supplied with 2 mM of CaCl_2_ (C.Cl). Aeration was skipped on the first day following transferred to the “split-root” condition. Root phenotypes were measured after 4 days of split-root treatment, with 12 roots analyzed for each condition. The maize roots were photographed, and the images were measured and analyzed using Gia Roots [30].

### Maize RNA-seq assay and analysis

For RNA-seq profiling, maize plants were harvested for RNA expression analysis 8 h after the treatments. Three RNA biological replicates were prepared for each condition. For the control plants (C.NO_3_ and C.Cl), a replicate of RNA sample was extracted from a pool of three seedlings (i.e. one shoot RNA sample from three seedlings, and one root RNA sample from six roots of three seedlings). For the heterogeneous N treated plants, a shoot RNA sample was extracted from a pool of six seedlings, while a root RNA sample was extracted from a pool of six roots from six plants. The tissues were harvested and flash frozen in liquid nitrogen. The samples were then ground to fine powder in liquid nitrogen using mortar and pestle. Total RNA was extracted from the tissue powder using mirVana miRNA Isolation Kit (Ambion, AM1560) with the Plant RNA Isolation Aid (Ambion, AM9690) following the manufacturer’s guide for total RNA extraction. The stranded RNA-seq libraries were made using the NEBNext^®^ Ultra™ II Directional RNA Library Prep Kit for Illumina^®^ (NEB cat E7768) and assessed using DNA high sensitivity D1000 ScreenTape system (Agilent cat 5067–5584). The RNA-Seq libraries were sequenced on an Illumina HiSeq 2500 v4 platform with 1 × 75 bp single-end read chemistry at the GenCore Facility at New York University Center for Genomics and Systems Biology. Raw reads were trimmed using BBDuk (v37.24) and then aligned to Zm-B73-REFERENCE-GRAMENE-4.014 using BBMap (v37.24) [35]. The mapped reads were assigned to genes by featureCounts (1.5.1) using AGPv4.32 [36]. For DEG analysis, the lowly expressed genes were first filtered out (the average CPM for two reps < 1) and the data was then normalized using the VSD function in DESeq2 (1.38.3) followed by voomWithQualityWeights function in limma (3.54.2) to accommodate the heterogeneity nature of split root data [33]. To identify N-supply and N-demand DEGs, we conducted pairwise comparison of Sp.NO_3_ vs. C.NO_3_ (FDR < 0.05) and Sp.Cl vs. C.Cl roots (FDR < 0.01 and |log_2_FC|>1). In the shoots, we also performed similar pairwise comparison used the same pipeline to identify DEGs between C.NO_3_ vs. Split (FDR < 0.05) and C.Cl vs. Split (FDR < 0.05).

### Across-species comparison

To identify the Arabidopsis homologs of tomato DEGs, the peptide sequences for the tomato genes were searched against the complete set of Arabidopsis peptides using blastp (E-value cutoff 1E-7)[37], and the highest hit was used as the most likely candidate of Arabidopsis homolog. The Arabidopsis homologs of maize DEGs were determined as previously described [38]. The gene lists from each species were compared using venny 2.1 [39] to find the overlaps among the three species. Pairwise gene set comparisons were performed using the genesect function in the virtual plant platform [26] to determine the significance of the overlap.

## Results

### Heterogeneous N environments experienced by roots trigger rapid transcriptomic responses in shoots of Arabidopsis

In this study, we aimed to detect early and temporal transcriptional events involved in response to heterogeneous N availability. To this end, we assayed N-dependent transcriptome responses from the shoots and roots of Arabidopsis (Col-0) seedlings placed in a split-root system exposed to heterogeneous N-supply. We monitored transcriptome responses at four early time points following N-supply vs. -deprivation, i.e. 0 h, 2 h, 4 h and 8 h (Fig. 1A), by RNA-Seq. These early time points were selected based on our previous study, which showed that the root transcriptome shifted from responding to the local N environment to systemic N signals within 8 h following the initiation of heterogenous N treatment [3].

Next, to identify shoot genes that are important for early systemic N-signaling, we determined DEGs in the shoots at 2, 4 and 8 h respectively (see Methods for details). At each time point, we compared three types of samples (Fig. 1A): (*i*) *CN*: (Control N) *shoots* from plants that are exposed to homogeneous N supply; (*ii*) *Split*: *shoots* from split-root plants that are exposed to heterogeneous N supply; and (*iii*) *C.Cl* (Control KCl) *shoots* from plants that are exposed to homogeneous N depletion. In analyzing the transcriptome responses to the various treatments, we tested the null hypothesis that the gene expression levels are unchanged among the three N conditions. We identified genes that are significantly differentially expressed among the CN, Split, and C.Cl conditions at 2 h (106 DEGs), 4 h (3,020 DEGs) and 8 h (4,293 DEGs) (Fig. 1B; Additional File 2 Table S1). More than 50% of DEGs are shared between neighboring time points, indicating a continuum of gene regulation over time (Fig. 1B). Furthermore, we compared the DEGs identified in this study with those reported by Poitout et al. [8], which analyzed shoot transcriptomes under CN and Split conditions [8] at the 24 h timepoint, and found overlaps with the later time points in our study (70 shared genes at 4 h and 114 at 8 h). To further characterize their roles in systemic N signaling, these DEGs were categorized based on their expression patterns using *post-hoc* Tukey’s test, which detected 11 *regulation groups* with distinct expression patterns across N conditions (Additional File 1 Fig. S1; Additional File 2 Table S1).

We were particularly interested in DEGs that are up-regulated or down-regulated in the shoots when the plants are exposed to heterogeneous N environment, compared to the control plants (regulation groups 1 to 5 in Additional File 1 Fig. S1; Additional File 2 Table S1). For example, groups 1 and 2 comprise genes that are induced in the split plants compared to the control plants; conversely, groups 3–5 include genes repressed in the split plants compared to the control plants (Additional File 1 Fig. S1). Collectively, these genes are termed “split-specific genes”. At 2 h, only three genes are “split-specific” (Additional File 1 Fig. S1; Additional File 2 Table S1). At the later time points (4 h and 8 h), more genes (63 and 75 genes) with “split-specific” patterns of expression are observed (Additional File 1 Fig. S1; Additional File 2 Table S1). In total, we identified 138 “split-specific” DEG in the shoots (group 1–5 in Table S1 in Additional File 2) whose expression level is significantly different in the heterogeneous N treated plants (“split”), compared to the homogeneous N treated plants (“CN” and “C.Cl”). This list comprises multiple genes encoding transcriptional regulators, including TFs MYB30 (AT3G28910; Fig. 1C), REM7 (reproductive meristem 7; AT3G18960; Fig. 1D), WOX2 (WUSCHEL related homeobox 2; AT5G59340; Fig. 1E), KNAT1 (KNOTTED like; AT4G08150; Fig. 1F), SPL7 (squamosa binding protein like 7; AT5G18830; Fig. 1G), and SCL11 (scarecrow-like TF 11; AT5G59460; Fig. 1H); as well as key players in phosphate signaling such as *PHO1* (AT3G23430) and *PHO2* (AT2G33770) (Fig. 1I and J) (40–42]. This suggests that specific signaling pathways are triggered in the shoots by the heterogenous N environment in the roots within as early as 2 h to regulate gene expression and coordinate nutrient status.

In addition, amongst the early N-responsive genes, we identified three regulation groups that are induced by N supply and/or repressed by N-depletion (groups 6–8 in Fig. S1 in Additional File 1), which are significantly enriched with biological processes “cellular nitrogen compound metabolic process” (Additional File 2 Table S2). Specifically, genes in group 7 showed an expression pattern where the transcript level is highest in “CN”, followed by “*split”*, and lowest in the “C.Cl” (Additional File 1 Fig. S1). This expression pattern tracks the total abundance of available N in the environment: two parts of 5 mM KNO_3_ for the C.NO_3_ plants; one part of 5 mM KNO_3_ for the split-root plants; and 0 mM KNO_3_ for the C.Cl plants (Fig. 1A). This regulation group includes N transport and assimilation genes such as nitrate transporter *NRT3.1*, nitrate reductase *NIA1*, nitrite reductase *NIR1*, and asparagine synthase *ASN2*, that exhibit an expression pattern reflecting the total external N abundance (Fig. 1K–N; Additional File 2 Table S1). This finding suggests that the plants can sense total N abundance within as soon as 2 h and adjust the expression level of key N assimilation genes accordingly to respond to environmental N availability. These early N availability responsive genes in group 7 are highly significantly enriched with the GO term “response to cytokinin stimulus” (Additional File 2 Table S2). Our results thus support a previous report that cytokinin responsive regulators are induced in proportion to global N levels in the shoots [3, 8].

Finally, we observed regulation groups 9, 10, and 11 whose expression levels are repressed by N supply and/or induced by N depletion (Additional File 1 Fig. S1). These groups include the gene encoding small peptide receptor kinase CEPR1 (Additional File 2 Table S1). This finding validates that the *CEPR1* is indeed induced by N starvation, as previously reported in several N foraging studies [10, 11].

### Roots displayed widespread transcriptomic response to the heterogeneous N condition

In the split-root system, roots serve as the primary responders, sensing and communicating local N conditions, interpreting shoot-borne systemic N signals, and promoting lateral root growth in N-rich patches. Thus, roots play a dual role as initiators and executors in N foraging. To probe the underlying molecular players, we compared the transcriptomes of four types of root samples: C.NO_3_ roots, Sp.NO_3_ roots, Sp.Cl roots, and C.Cl roots (Fig. 1A), at 0, 2, 4, 8 h after the onset of N treatments. Our statistical analysis of the transcriptome responses to the various treatments used the null hypothesis that the gene expression levels are unchanged among the various N conditions. We identified 505, 752, and 915 genes that are significantly differentially expressed at 2, 4, and 8 h separately (Additional File 1 Fig. S2; Additional File 2 Table S3). These DEGs were further classified to multiple regulation groups by distinct expression patterns (indicated by “regulation group number” in Additional File 2 Table S3). Based on GO term enrichment analysis, these DEGs are significantly enriched (FDR < 0.05) with multiple biological processes relevant to N metabolism, including “nitrate assimilation”, as well as energy metabolism, transcription and translation, ion transport, and response to cytokinin (Additional File 1 Fig. S2; Additional File 2 Table S4). We were specifically interested in genes responding to systemic “N-demand signals” as defined in Ruffel et al. [3], which are differentially expressed between C.NO_3_ and Sp.NO_3_ roots, and identified 30, 59, and 89 such genes from 2 h, 4 h and 8 h separately. This DEG list includes nitrate transporters (*AT5G60770* and *AT1G12940*) and auxin signaling genes (*AT5G20820* and *AT1G23160*) (Additional File 2 Table S5), consistent with their roles in taking up nitrate and stimulating root growth in N-rich patches, respectively. Similarly, we identified 185, 107, and 185 genes responding to systemic “N supply signals” (that are differentially expressed between C.Cl and Sp.Cl roots)” as defined in Ruffel et al. [3] at 2 h, 4 h and 8 h separately, which are enriched with functional groups such as energy metabolism and rRNA processing (Additional File 2 Table S5).

### Inter-organ correlated gene network provides systems-level insights into root-shoot-root signaling during root N-foraging

**Fig. 2:**
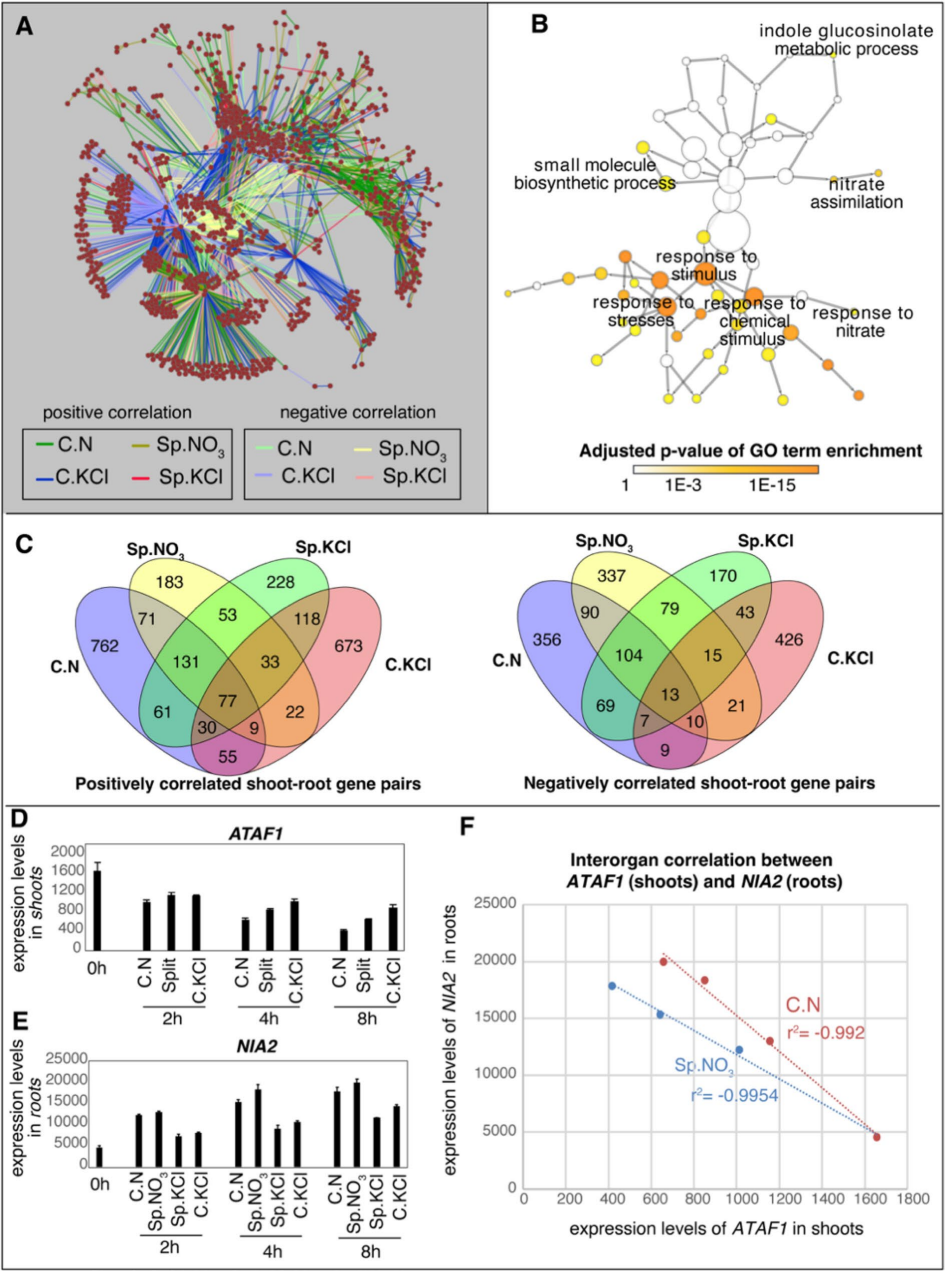
Inter-organ correlation analysis identified gene pairs with likely roles in long-distance signaling. **A** The inter-organ correlation network includes all gene pairs of shoot gene X and root gene Y whose time course expression profile showed significant correlation. The color of edges indicates the specific N condition where the correlation was identified, as well as the direction of the correlation (positive of negative). The correlation analysis was performed in R with rcorr function. The p-values were then corrected for multi-testing error using the p.adjust function (FDR method) in R. An FDR cutoff of 0.001 was selected to be an ideal cutoff based on an FDR sensitivity test. **B** Gene Ontology (GO) network showing the enrichment of GO functional categories among the genes showing significant inter-organ correlation. Each node is a GO term. The color of the node represents the significance of enrichment for each GO term. Related GO terms are connected by edges. The network was generated using the BINGO package in Cytoscape based on the inter-organ correlated network in **A**. **C** Venn diagrams showing the overlaps of inter-organ correlated gene pairs identified under different N conditions for positively or negatively correlated gene pairs, separately. One such gene pair is highlighted (**D**–**F**), including *ATAF1* expressed in the shoots (**D**) and *NIA2* expressed in the roots. Their expression levels in the shoots or roots as measured by RNA-seq were plotted (**D**&**E**) as in Fig, 1, and a strong correlation between the shoot expression of *ATAF1* and root expression of *NIA2* was observed under the Sp.NO_3_ condition and C.NO_3_ condition (**F**), in agreement with a N-supply signaling across organs

It has been proposed that a long distance root-shoot-root signal relay mediates root foraging responses in heterogenous N environments [3]. We reasoned that part of this long distance systemic N-signaling could involve a shoot-borne transcript (“mRNA *X*”) trafficking to roots, where it causes changes in the levels of a root-borne transcript (“mRNA *Y*”), or vice versa [25]. To identify such shoot–root transcript pairs (“mRNA *X* and *Y*”), whose expression level may exhibit correlation across organs due to the long-distance influence, we performed a three-step data analysis: (*i*) first, we calculated the correlation of transcript levels measured over time in our study between any gene pairs (gene *X* and gene *Y*), where transcript levels of gene *X* is measured in the shoots while the gene *Y* is measured in the roots of the same plants (i.e. CN shoots with C.NO_3_ roots, split shoots with Sp.NO_3_ roots, split shoots with Sp.Cl roots, and C.Cl shoots with C.Cl roots in Fig. 1A). We identified gene pairs where the time-course expression of a gene in the shoots is highly significantly correlated with the expression levels of another gene in the roots of the same plant (threshold of FDR adjusted *p* < 0.001), which indicates a potential regulatory relationship between the two genes; (*ii*) next, to select gene pairs that are relevant to N responses, the following filter was applied: in each correlated gene pair, the shoot gene must be differentially expressed across different N conditions in the shoots, and the root gene must be differentially expressed across N conditions in roots in our study; (*iii*) finally, to leverage information of previously identified shoot–root trafficking RNAs, we intersected identified gene pairs with experimentally-determined trafficking RNAs [25], and selected gene pairs where at least one transcript was reported to travel between shoots and roots. Overall, this across-organ mRNA analysis pipeline identified 1,854, 1,288, 1,231, and 1,561 gene pairs whose mRNAs travels from root to shoot (or vice versa) for C.NO_3_, Sp.NO_3_, Sp.Cl and C.Cl conditions, separately (Additional File 2 Table S6). Collectively, these cross-organ interactions can be viewed as an integrated network of 1,064 nodes (representing genes/transcripts) and 5,934 edges (representing inter-organ correlations) (Fig. 2A), where a single gene may be involved in several pairs. This network is enriched with functional categories such as response to chemical stimulus (nitrate) and metabolic process such as nitrate assimilation, as determined by BINGO (Fig. 2B) [29].

We were most interested in gene pairs whose inter-organ correlation were identified under specific N conditions. For example, if an N supply condition triggers root-to-shoot signaling, we expect to observe correlated shoot–root gene pairs between CN shoots and C.NO_3_ roots, as well as between shoots of split plants and Sp.NO_3_ roots, as both conditions involve sensing available N supply. Similarly, if there is split-root specific shoot-to-root signaling, we expect to identify the relevant shoot–root gene pairs between the split shoots and Sp.NO_3_ roots, as well as between the split shoots and Sp.Cl roots. Therefore, we compared the gene pairs identified under the four different N conditions using Venn diagram (Fig. 2C; Additional File 2 table S6), and detected condition-specific gene pairs. One interesting gene pair is *ATAF1* (*AT1G01720*) in the shoots and *NIA2* (*AT1G37130*) in the roots. *ATAF1* encodes a No Apical Meristem (NAM) domain TF responsive to stresses and important for regulating plant growth [43], and it is induced by N limitation in our study (Fig. 2D). *NIA2* encodes a nitrate reductase essential for N assimilation and was observed to be induced by local N supply (Fig. 2E). Interestingly, the mRNA level of *NIA2* in the roots is negatively correlated with the mRNA level of *ATAF1* in the shoots (Fig. 2F), under C.NO_3_ (between CN shoots with C.NO_3_ roots) and Sp.NO_3_ conditions (between split shoots with Sp.NO_3_ roots). The *ATAF1* mRNA was shown to transport from shoots to roots, and the *NIA2* mRNA was shown to transport from roots to shoots [25]. Collectively, this suggested a potential inter-organ signaling event where *NIA2* transcripts are induced in the roots by N-supply, and travel from roots to shoots and consequently repress *ATAF1.* Or alternatively, *ATAF1* transcript could be induced by N-depletion, and travels from shoots to roots to repress *NIA2* expression. Overall, our inter-organ correlation analysis identified thousands of transcripts that potentially travel from shoots to roots, or vice versa, to affect gene transcription in the remote organ in a N-dependent manner.

### Histone methyltransferase SDG8 mediates heterogenous N response

**Fig. 3:**
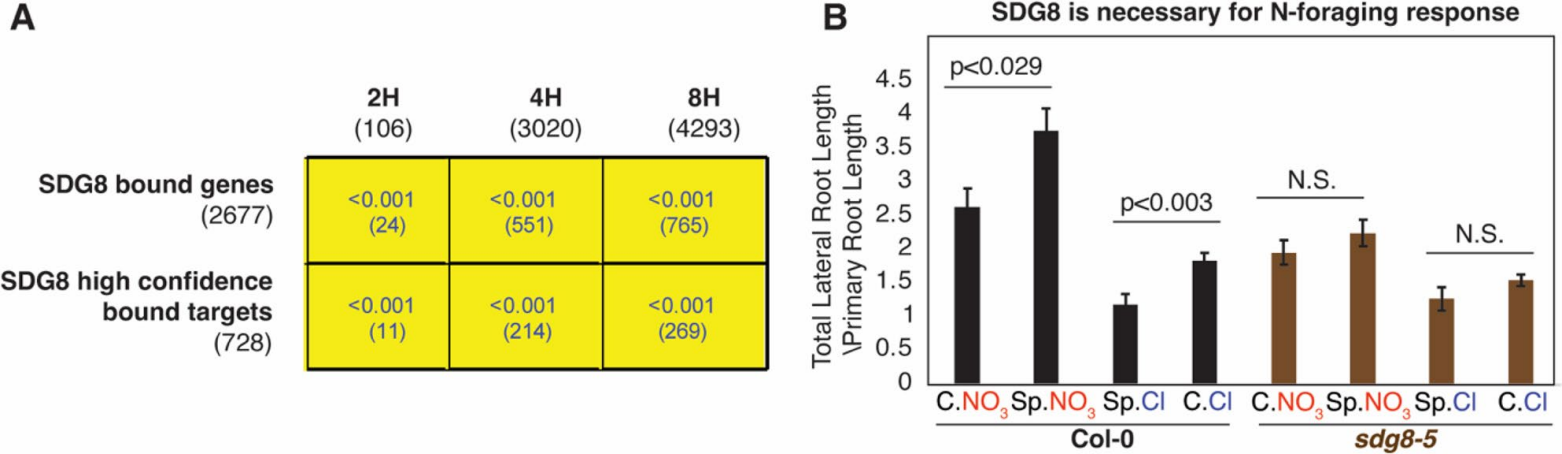
Histone methyltransferase SDG8 are involved in the mediating the root foraging for N. **A** A GenSect plot showing gene set comparisons between SDG8 genomic targets (represented by rows) and the differential expressed genes in shoots identified in this study (represented by columns). The 2,677 “SDG8 bound genes” were identified previously through SDG8 ChIP-seq; and the 728 “SDG8 high confidence bound target” were previously reported as genomic targets whose transcript levels and histone acetylation levels (H3K36me3) were dependent on SDG8. Each cell represents the overlap between two gene sets indicated by the corresponding row and column. The two numbers in the cell represent the significance of the overlap (e.g. *p* < 0.001), as determined by the GenSect function of the Virtual Plant platform (see method for details), and the number of genes overlapped (in parenthesis). Overall, significant overlaps were observed among the genomic targets of SDG8 and the differentially expressed genes in our split-root study, suggesting that SDG8 may mediate the differential gene expression changes in the heterogeneous N responses. **B** The wild-type (Col-0) showed active root foraging phenotype, evidenced by a significantly enhanced lateral root growth in the Sp.NO_3_ condition compared to the C.NO_3_, as well as a reduced lateral root growth in the Sp.Cl compared with the C.Cl condition. In contrast, the *sdg8-5* mutant loses such lateral root growth responses to the heterogeneous N conditions. This finding supports that SDG8 is required for the normal root foraging for N. The phenotyping was performed as described in methods. The mean across 8–13 individual seedlings was plotted, and the error bars represent standard error of the mean. The pvalues were determined by standard student’s t-test

While specific hormone and TF regulators have been shown to regulate the root foraging phenotype [3, 7, 8], a possible role for chromatin regulators in mediating the root foraging behavior has been unexplored. Recently, the histone methyltransferase SDG8 was discovered to mediate N response in Arabidopsis [16]. Therefore, we tested whether SDG8 also contributes to root foraging responses. To this end, we compared the 728 genomic targets of SDG8 previously reported by [44] and the 2,677 SDG8-bound targets reported in [45], with the DEGs regulated in response to split-root conditions in our study. We focused on the DEGs identified from the shoots, because the published SDG8 targets were identified from whole seedlings, which consist primarily of shoot tissues. We found that both sets of reported SDG8 targets have significant overlaps with the genes regulated under heterogeneous N environments (Fig. 3A), suggesting that SDG8 might play a role in regulating N-responsive genes in the split-root system. To further test it, we examined the root foraging behavior of a *sdg8-5* mutant previously characterized [16] using the split-root system, and found that the *sdg8-5* mutant loses the ability to respond to either systemic N-demand signal (C.NO_3_ vs. Sp.NO_3_ roots) or systemic N-supply signal (Sp.Cl vs. C.Cl roots), while it retains the regulation of root development response to local NO_3_^−^ supply (Fig. 3B), supporting an essential role for SDG8 in regulating root forage response to systemic N signals.

### Cross-species comparison of root N-foraging responses uncovered conserved and/or unique response to heterogeneous N conditions

To determine whether the N foraging mechanisms were conserved across divergent plant species, we additionally examined the root foraging phenotypes and corresponding transcriptomes of crop models tomato (*Solanum lycopersicum*) and maize (*Zea mays*), which were then compared with our observations from the model plant Arabidopsis.

#### Tomato cultivars M82 and Heinz 1706 showed distinct root foraging phenotypes and gene expression

**Fig. 4:**
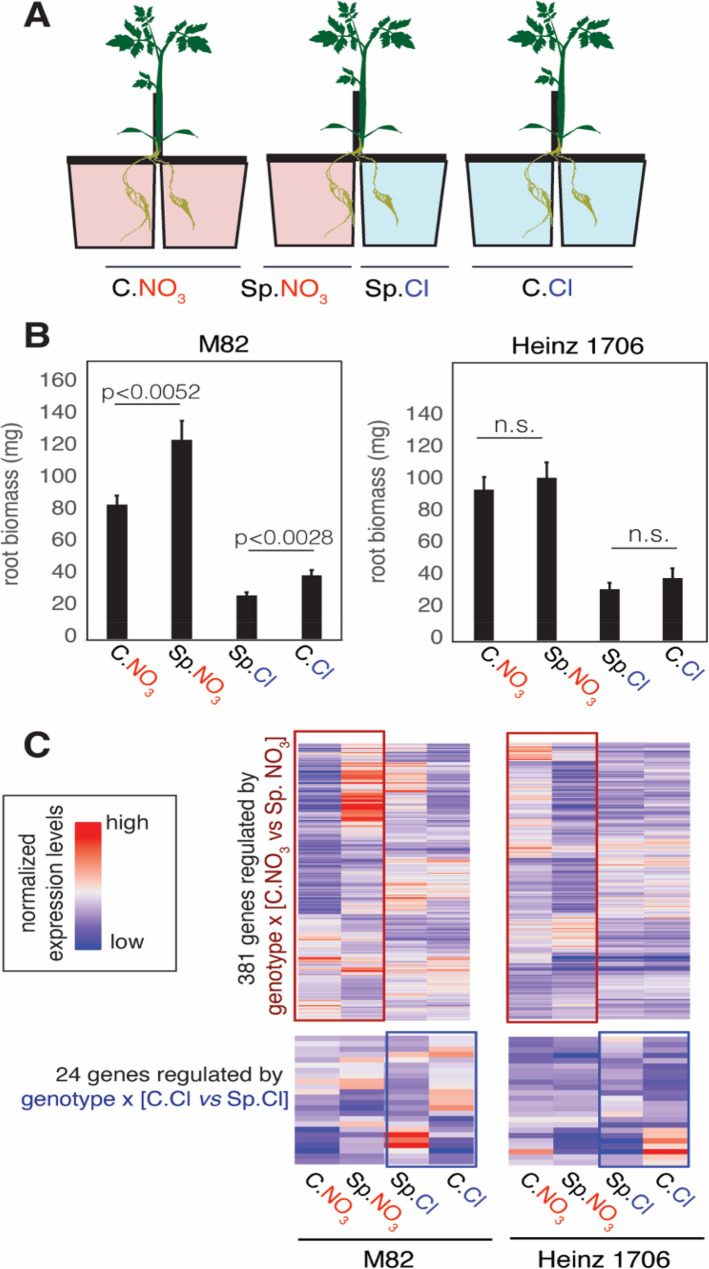
Different tomato cultivars displayed distinct root foraging phenotype and gene expression. **A** Schematic illustration of the hydroponic split-root setup used to investigate root foraging behavior in tomato plants. **B** Two cultivars, M82 and Heinz 1706, were tested in split-root systems. M82 exhibited an active root foraging phenotype, as indicated by significantly enhanced root growth under the Sp.NO₃ condition compared to C.NO₃, and reduced root growth under Sp.Cl compared to C.Cl. In contrast, Heinz 1706 did not show significant responses under either condition, suggesting that M82 is a more active forager than Heinz 1706. The mean was plotted with *N* = 12. Error bars represent standard error of the mean. The p-values were determined by student’s t-test. **C** Heatmap showing the root expression levels of genes differentially expressed between the two cultivars in response to systemic N signals. Expression patterns of genes responsive to the systemic N-supply signal (genotype × N-supply interaction; C.Cl vs. Sp.Cl) are highlighted in the blue box, while genes responsive to the systemic N-demand signal (genotype × N-demand interaction; C.NO₃ vs. Sp.NO₃) are highlighted in the red box

To investigate N foraging of tomato plants, we established a split-root hydroponic system. Briefly, tomato seeds were germinated and transferred to hydroponic systems containing full nutrient media and grown to ~ 3-week-old seedlings. The roots were then trimmed to keep only two lateral roots of equal length, which developed into two root systems. After a 4 d recovery from the trimming, the plants were transferred to N-starvation media for 2 days, followed by moving to a hydroponic split-root setup (Fig. 4A). We evaluated two cultivars, M82 and Heinz 1706, that are commonly used for physiological and molecular studies [31, 46]. Interestingly, the M82 displayed active root foraging, evidenced by the significant difference between the heterogeneous N-supplied condition (Sp.NO_3_) with the homogeneous N-supplied condition (C.NO_3_), as well as between the heterogeneous N-depleted condition with the homogeneous N-depleted condition (Sp.Cl vs. C.Cl), measured through root biomass (Fig. 4B). By contrast, the Heinz 1706 variety does not show any prominent root foraging behavior, only displaying a response to local N availability (Fig. 4B). In summary, we found that the M82 and Heinz 1706 cultivars display distinct root foraging capacity for N.

To identify the molecular mechanisms underlying the divergent foraging behaviors of M82 and Heinz 1706, we profiled the shoots and root transcriptomes of both cultivars after 6 h of split-root treatment. We focused on root genes whose expression levels changed differently between the two cultivars, driven by the systemic N-supply signal (i.e. interaction of genotype x N-supply treatment [C.Cl vs. Sp.Cl]), or systemic N-demand signal (i.e. interaction of genotype x N-demand treatment [C.NO_3_ vs. Sp.NO_3_]), determined by DESeq2 [33]. We detected 381 genes that are differentially expressed between the C.NO_3_ and Sp.NO_3_ roots in a genotype-dependent manner (Fig. 4C; red boxes; Additional File 2 Table S7). These genes are potentially involved in the systemic N-demand signaling, which promotes root foraging responses in M82, but is misregulated in Heinz 1706. These genes are involved in N metabolism (ASN1 [Solyc06g007180] and AMT1 [Solyc09g090730]), central metabolism signaling (bZIP53 [Solyc01g100460] and KIN10 [Solyc03g115700]), as well as root growth (expansin [Solyc12g089380] and auxin signaling [Solyc03g121060, Solyc01g097290, Solyc04g056620, and Solyc02g082450]) (Additional File 2 Table S7). Similarly, we identified 24 genes differentially expressed between the C.Cl and Sp.Cl roots in a genotype-dependent manner, indicating their potential relevance with the response to systemic N-supply signaling (Fig. 4C; blue boxes; Additional File 2 Table S7). Moreover, in the shoots, we identified 642, 108 and 194 genes that are differentially expressed between C.NO_3_ vs. C.Cl, C.NO_3_ vs. split, or C.Cl vs. split conditions in a genotype-dependent manner (Additional File 2 Table S7). Collectively, these differentially expressed genes provide molecular insights into the distinct root foraging phenotypes of the two tomato cultivars.

#### Maize plants displayed classic root N-foraging response

**Fig. 5:**
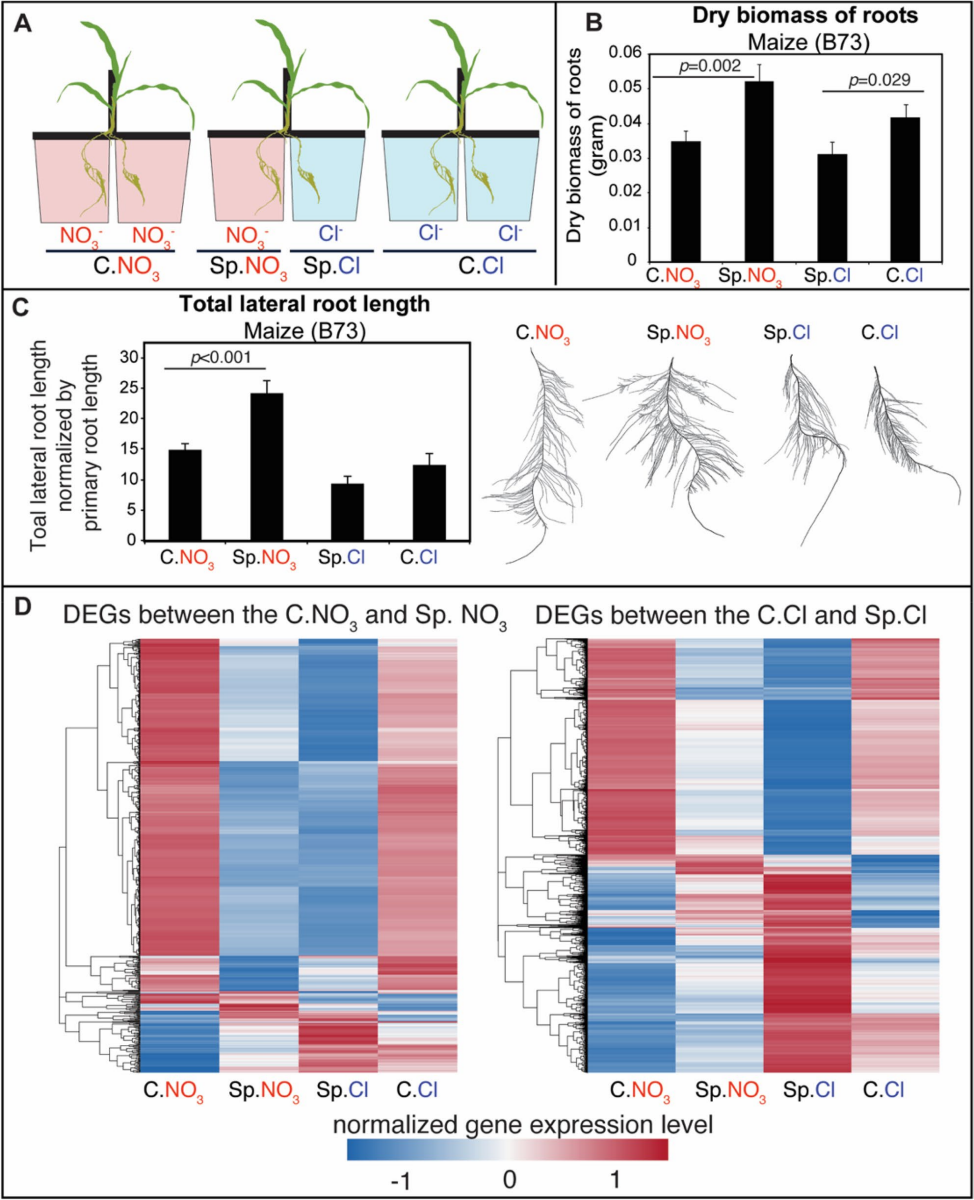
Split-root nitrogen treatment triggers root foraging phenotype and altered gene expression in maize seedlings. **A** Schematic illustration of the hydroponic split-root setup used to investigate root foraging behavior in maize seedlings. **B** Maize (B73) seedlings exhibited an active root foraging phenotype, as indicated by significantly enhanced root biomass under the Sp.NO₃ condition compared to C.NO₃, and reduced root biomass under Sp.Cl compared to C.Cl. The mean was plotted with *N* = 12. Error bars represent standard error of the mean. The p-values were determined by student’s t-test. **C** The root foraging is also evidenced by the measurement of total lateral root length, with significantly enhanced root growth under the Sp.NO₃ condition compared to C.NO₃. Exemplary root images were included. The mean was plotted with *N* = 12. Error bars represent standard error of the mean. The p-values were determined by student’s t-test. **D** Heatmap showing the root expression levels of genes differentially expressed between C.NO₃ vs. Sp.NO₃ (hence responding to the systemic N-demand signal), and that differentially expressed between C.Cl vs. Sp.Cl (hence responding to the systemic N-supply signal)

To study root foraging in maize (*Zea mays* cultivar B73), we performed a split-root experiment using a protocol similar to that described for tomato (see methods) (Fig. 5A). Measured by dry root biomass (Fig. 5B) and total root length (Fig. 5C), we observed a consistent response to the N-demand signal (difference between C.NO_3_ and Sp.NO_3_ roots). To probe the underlying mechanism, we profiled shoot and root transcriptomes 8 h after the split-root treatment, and identified 661 root DEGs with differential expression between the C.NO_3_ and Sp.NO_3_ conditions (Fig. 5D; Additional File 2 Table S9A), which are potentially relevant to the observed root growth difference between C.NO_3_ and Sp.NO_3_ roots. In addition, we identified 10,248 DEGs between C.Cl and Sp.Cl roots (Fig. 5D), 4,281 DEGs between C.NO_3_ shoots vs. split shoots, and 289 DEGs between C.Cl shoots vs. split shoots (Additional File 2 Table S9B).

#### Comparison of transcriptomic responses to heterogenous N supply across Arabidopsis, tomato, and maize uncovered conserved and unique molecular underpinnings

To investigate if conserved mechanisms mediate root N-foraging across different plant species, we compared the root DEGs among Arabidopsis, tomato, and maize identified in our study. We focused on comparing root DEGs because genes responding to systemic N signals can be clearly defined in this organ by comparing heterogeneous N-conditions with the corresponding homogeneous N-treated controls. To facilitate comparisons of similar time points and root foraging phenotypes, we chose or generated the following gene lists from our study for comparison: (i) for the model Arabidopsis, we used the root DEGs detected at the 8 h timepoint, which were differentially regulated between Sp.NOmod_3_ and C.NO_3_ (responding to the N-demand signaling), or between Sp.Cl and C.Cl roots (responding to the N-supply signal) (Fig. 1A; Additional File 2 Table S3); therefore, the Arabidopsis and maize were sampled at 8 h, while tomato sampling were performed at 6 h, providing reasonably comparable sampling windows for cross-species analysis. (ii) for the crop tomato, we re-analyzed the RNA-seq data for the M82 cultivar only (as this cultivar shows active root foraging [Fig. 4A]). As in Arabidopsis, we included the genes that were differentially expressed between Sp.NO_3_ and C.NO_3_ roots (responding to the N-demand signaling; 236 genes in Additional File 2 Table S8), and between Sp.Cl and C.Cl roots (responding to the N-supply signal; 14 genes in Additional File 2 Table S8), at 6 h after split-root treatment; (iii) for maize, we included the root genes that are differentially expressed between Sp.NO_3_ and C.NO_3_ (responding to the N-demand signaling), and between Sp.Cl and C.Cl (responding to the N-supply signal) at 8 h after the split-root treatment (Additional File 2 Table S9).

**Fig. 6:**
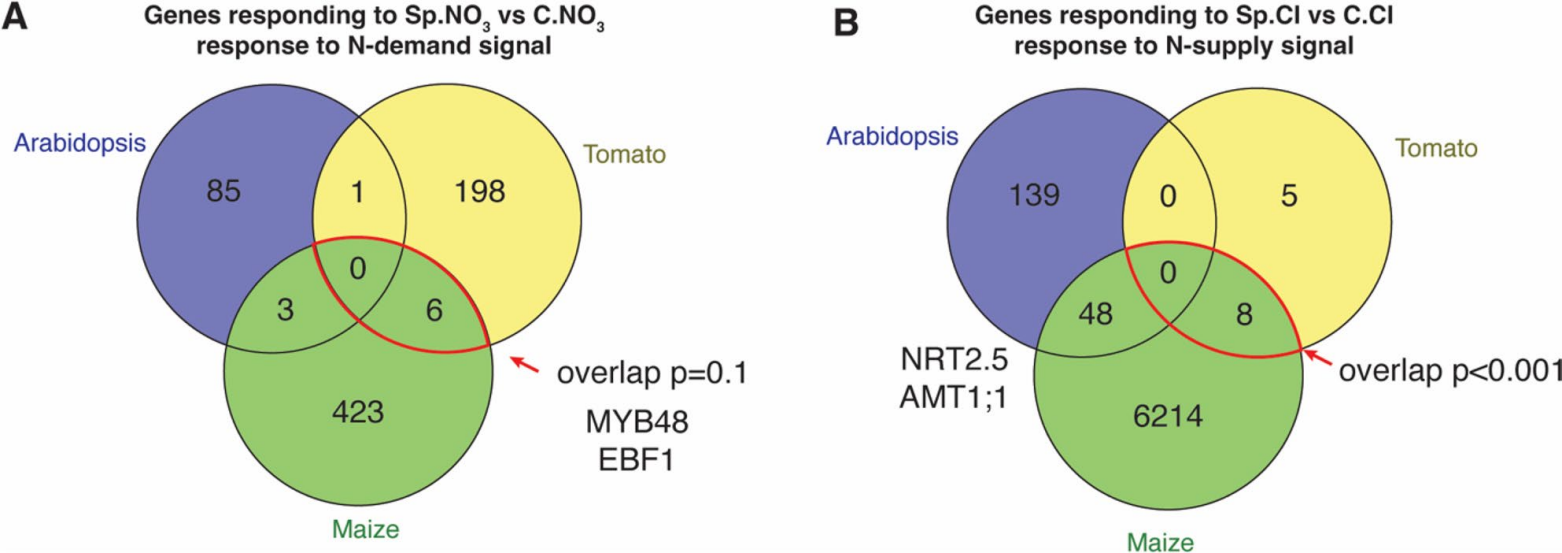
Distinct and shared genes mediated the systemic N signaling across different species. **A** Venn diagram comparing genes differentially expressed in response to systemic N-demand signaling (Sp.NO₃ vs. C.NO₃) across Arabidopsis, tomato, and maize roots. A modest overlap (*p* = 0.1) was detected between tomato and maize, including shared transcriptional regulators such as MYB48 and EBF1, suggesting limited but potentially functionally important convergence. The significance of overlap was determined using the Genesect function in the VirualPlant platform. **B** Venn diagram comparing genes responsive to systemic N-supply signaling (Sp.Cl vs. C.Cl) in the three species. A significant overlap (*p* < 0.001) was observed between tomato and maize, indicating a more conserved transcriptional response to N sufficiency across these two crop species. Arabidopsis exhibited minimal non-significant overlap with either species in both conditions, but its overlap with maize includes known N metabolism genes such as nitrogen transporters NRT2.5 and AMT1;1

Interestingly, we did not identify any genes regulated by the same systemic N signal across all three species (Fig. 6). However, we observed a significant overlap between tomato and maize in genes regulated by the systemic N-supply signal (Fig. 6B), as well as a modest yet notable overlap in genes regulated by the systemic N-demand signal (Fig. 6A), including TFs belonged to the MYB or EBF families (Fig. 6A).

## Discussion

The ability of plants to forage in the soil for mineral nutrients, especially nitrogen (N), is vital to their growth, reproductive success, and agricultural nutrient use efficiency. Here, we used the split-root system to study systemic signaling that directs root foraging in response to a heterogenous N supply. Specifically, our study generated and analyzed comprehensive transcriptomic datasets, both longitudinally (early time-course) and in scope (across shoots and roots in three plant species), to investigate underlying molecular mechanism of root foraging. In the model plant Arabidopsis, we profiled the transcriptomes in shoots and roots at 0, 2, 4, and 8 h after the heterogenous N treatment. We discovered that roots sense the heterogenous N environment, which leads to changes in gene expression in the shoots within 2 h (Fig. 1). To interrogate inter-organ signaling, we constructed an inter-organ gene correlation network to identify shoot and root transcripts that function collaboratively over long distance to mediate root foraging (Fig. 2). A recent study using a similar approach to identify trans-organ co-expression regulatory network, but with different algorithms, identified TGA7 as a long distance regulator [47]. The shoot expression level of *TGA7*, encoding a bZIP family TF, was associated with N-deficient response in roots [47]. Furthermore, TGA7 protein was found to travel from shoots to roots and regulate different target genes in the two organs, likely to coordinate shoot–root growth under changing N conditions [47]. Similarly, in our analysis, we also detected transcript pairs that showed correlation in temporal expression across organs, indicating potential long-distance regulation. We further filtered these transcript pairs by requiring that the shoot- or the root-borne transcripts had been previously reported to travel from shoot-to-roots, or root-to-shoot [25], although it is worth noting that the identification of such inter-organ trafficking mRNAs may suffer from false positivity according to a recent study [48]. Overall, our analysis identified thousands of transcript pairs that may function across long distance between shoots and roots to mediate heterogenous N responses. The possible underlying mechanisms is that a shoot-borne mRNA travels via phloem to the roots, where it is translated into a regulatory protein (such as TF, kinase, posttranscriptional regulatory proteins, etc.) to control the expression of downstream genes in the roots [49]. Similarly, a root-borne mRNA could travel via xylem to shoots, translated into proteins, and regulate gene expression in the shoots. This could represent a fundamental mechanism that coordinates metabolic and developmental states across two distal organs, in addition to other mechanisms such as the long-distance transport of small RNAs (50–52], proteins [53, 54], peptides [11], metabolites [55], and hormones [8, 34]. The extent to which this mechanism contributes to root foraging for N requires further experimental validation, such as grafting assays.

Chromatin modifications have the potential to serve as an interface between environmental fluctuations and gene expression [56]. We previously reported that the histone methyltransferase SDG8 deposits permissive H3K36me3 modification at select gene loci in response to N changes [16]. In the current study, we found a significant overlap between the reported genomic targets of SDG8 and the DEGs regulated in our split-root experiments (Fig. 3), indicating a potential role of SDG8 in mediating the observed gene expression changes. In support of this, we found that SDG8 was indeed required for the wild-type like root foraging phenotype, as the *sdg8* mutant lose the ability to respond to systemic N-signaling (Fig. 3). Our prior work primarily uncovered the molecular function of SDG8 in N responses in the shoots [16], therefore, it is tempting to speculate that SDG8 acts in the shoots to affect root foraging. However, it is also possible that SDG8 plays a regulatory role locally in the roots, as it has been reported to be induced by N in specific cell layers in the roots [57]. Overall, chromatin level regulation may be uniquely suited to integrate systemic and local metabolic signals: chromatin modifying machinery can deposit histone marks at relevant gene loci (e.g., encoding transporters or enzymes in N metabolism) to “label” the status of one input (e.g., a local signal), thereby modulating the transcriptional response of that gene locus to another input (e.g., systemic signal).

The observation of similar root foraging behavior across Arabidopsis, tomato (Fig. 4) and maize (Fig. 5) supports that root foraging is a conserved phenomenon across species. Surprisingly, while the physiological phenotype is similar across species, our data suggested that wild and cultivated species might rely on different molecular circuits for achieving the differential root growth. Maize was domesticated about 9000 years ago [58], and tomato was domesticated about 7000 years ago [59]. The past several thousand years of artificial selection and breeding have taken place on topsoil enriched with N-input since prehistoric times [60]. As a result, the genetic selection of crops such as maize and tomato has been likely shaped by N-improved soils, in contrast to wild species like Arabidopsis, potentially leading to distinct genetic circuits fixed for foraging for high-N patches. Highlighting the potential influence of breeding, maize and tomato share significant overlaps of DEGs associated with systemic N responses, despite the dicot-monocot split ~ 200 million years ago (Fig. 6). This overlap is more prominent than the overlap between Arabidopsis and tomato that are both dicot species diverged ~ 130 million years ago (Rosids vs. Asterids) [61] (Fig. 6), highlighting the possible convergent rewiring of N-responsive gene networks during crop domestication. The shared genes between tomato and maize for systemic N signaling include two transcriptional regulators (MYB and EBF). Specifically, the *EBF* gene is upregulated in the split-N (Sp.N) roots compared to control-N (C.N) roots in the actively foraging tomato cultivar M82 (fold change = 1.52, significantly regulated), as well as in maize B73 (fold change = 1.4, significantly regulated). In contrast, the *EBF* is not significantly regulated in the non-foraging tomato cultivar Heinz 1706 (fold change = 1.0) or in Arabidopsis that actively forages for N but has never been selected for breeding (fold change = 1.1).

*EBF* encodes an EIN3-binding F-box protein that mediates the targeted degradation of EIN3, a central regulator of ethylene signaling. By promoting EIN3 degradation, EBF attenuates ethylene responses [62]. Because ethylene is known to repress root growth [62], the upregulation of *EBF* in M82 and B73 roots under Sp.N conditions may reduce ethylene signaling, thereby allowing enhanced root growth in N-rich patches. This regulatory pattern is not observed in Arabidopsis, suggesting a divergence in the molecular circuit underlying root N foraging. Ethylene plays a central role in abiotic stress responses, and stress tolerance and nutrient use efficiency are key traits selected during crop domestication and breeding. Therefore, the differential regulation of *EBF* may help explain differences between wild and cultivated species, where selection for nutrient use efficiency and yield may have reshaped the trade-offs between environmental responsiveness and growth, ultimately contributing to distinct molecular mechanisms governing root N foraging.

Interestingly, cultivar level variation was observed in tomato (Fig. 4), also supporting the notion that artificial selection through breeding may have influenced root foraging traits. Indeed, the comparative transcriptomic analysis (Fig. 4) provides molecular insight into the differential N foraging behavior observed between Heinz 1706 and M82. Genes that are specifically regulated under heterogeneous N conditions (Sp.NO₃ vs. C.NO₃) in M82, but show weaker or opposite regulation in Heinz 1706, may represent key molecular determinants underlying this phenotypic divergence (Fig. 4C). Notably, several auxin transport and signaling genes (Solyc03g121060, Solyc01g097290, Solyc04g056620, and Solyc02g082450) were differentially regulated, including two auxin efflux transporters and two IAA family members. These observations suggest that M82 may retain the capacity to dynamically modulate auxin signaling in response to systemic N signals to regulate root growth, whereas Heinz 1706 has a reduced response. This difference could arise from variation at any level of the signaling pathway, including N sensing, signal transduction, or downstream transcriptional regulation. It will be interesting to further investigate the mechanistic basis of this divergence and to determine whether it is linked to the breeding history of Heinz 1706 for the processing industry.

Moreover, we also cannot rule out the possibility that the similarity observed between maize and tomato but not Arabidopsis are driven by technical differences, such as the split-root experimental setup, or affected by inherent differences in the size of plants. A previous transcriptomic comparison between Arabidopsis and Medicago, although at much later time points (days), also revealed a limited overlap of differentially expressed genes [6]. To fully support this hypothesis, more comprehensive comparisons including multiple cultivated crops and their wild relatives, across different clades of plant evolution, are needed.

## Conclusion

Overall, our study provides new insights into the molecular basis of root nitrogen foraging. We found that root foraging for N involved early interorgan signaling events triggered by heterogeneous N supply, as well as a previously unrecognized role of chromatin regulation. We also found that both model and crop species exhibit a conserved root N-foraging growth response, with genotype-specific variation, that are likely mediated by partially conserved, partially unique molecular circuits.

## Supplementary Information

Supplementary Material 1.

Supplementary Material 2.

## Data Availability

The RNA-seq datasets described in the study are deposit in NCBI SRA with accession number PRJNA1261883.
