# Supplementary material for: Uncovering the early and conserved molecular mechanisms of root nitrogen foraging in model and crops
Source: BMC Genomics. 2026 Mar 10;27:384. doi: 10.1186/s12864-026-12736-5 (PMC13085526; doi:10.1186/s12864-026-12736-5)
Supplement: Supplementary file 2 — Supplementary Material 2. [file 12864_2026_12736_MOESM2_ESM.docx]

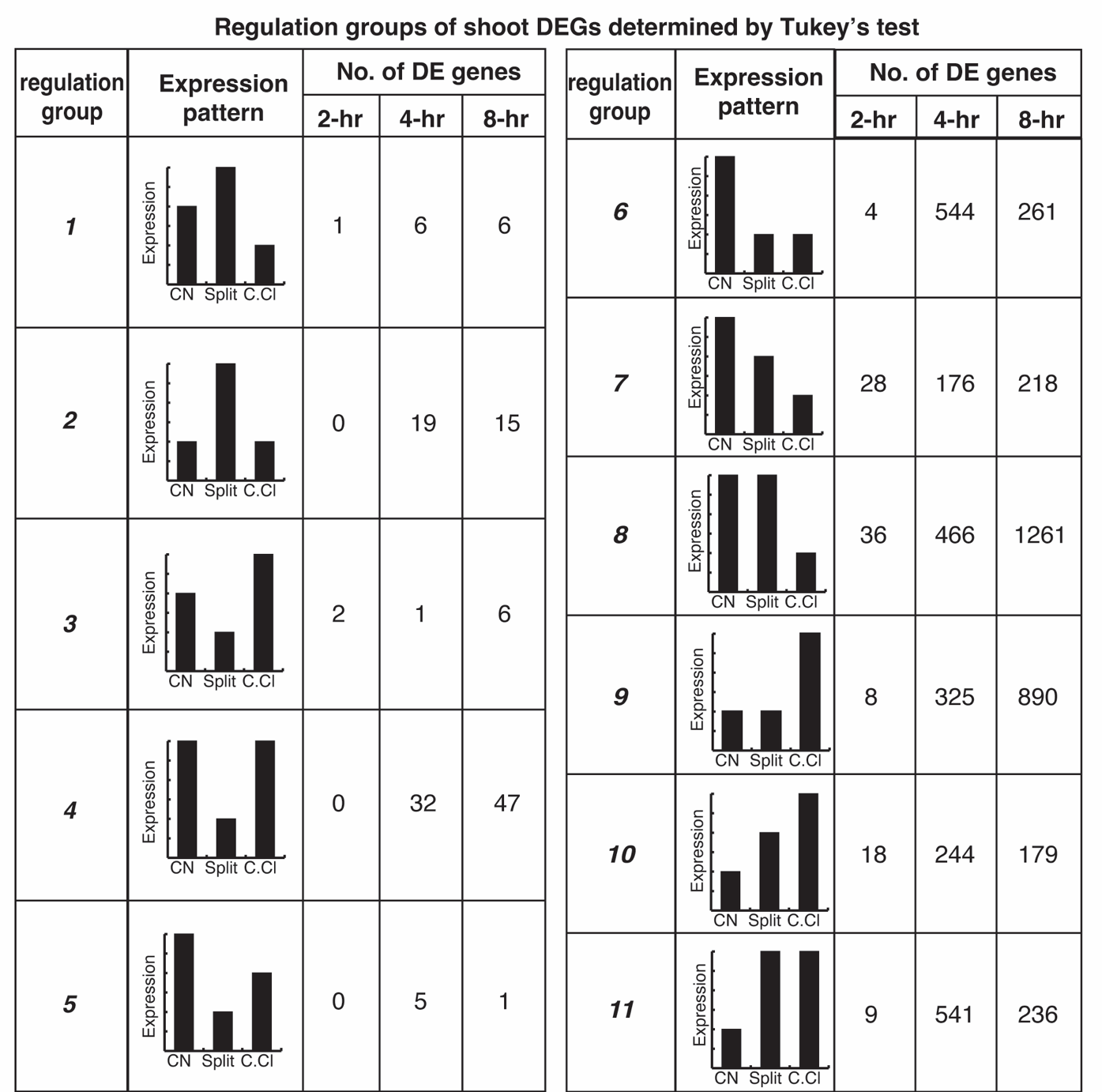


**Supplemental Figure S1. Expression patterns and number of genes for each regulation group among shoot DEGs in Arabidopsis.**


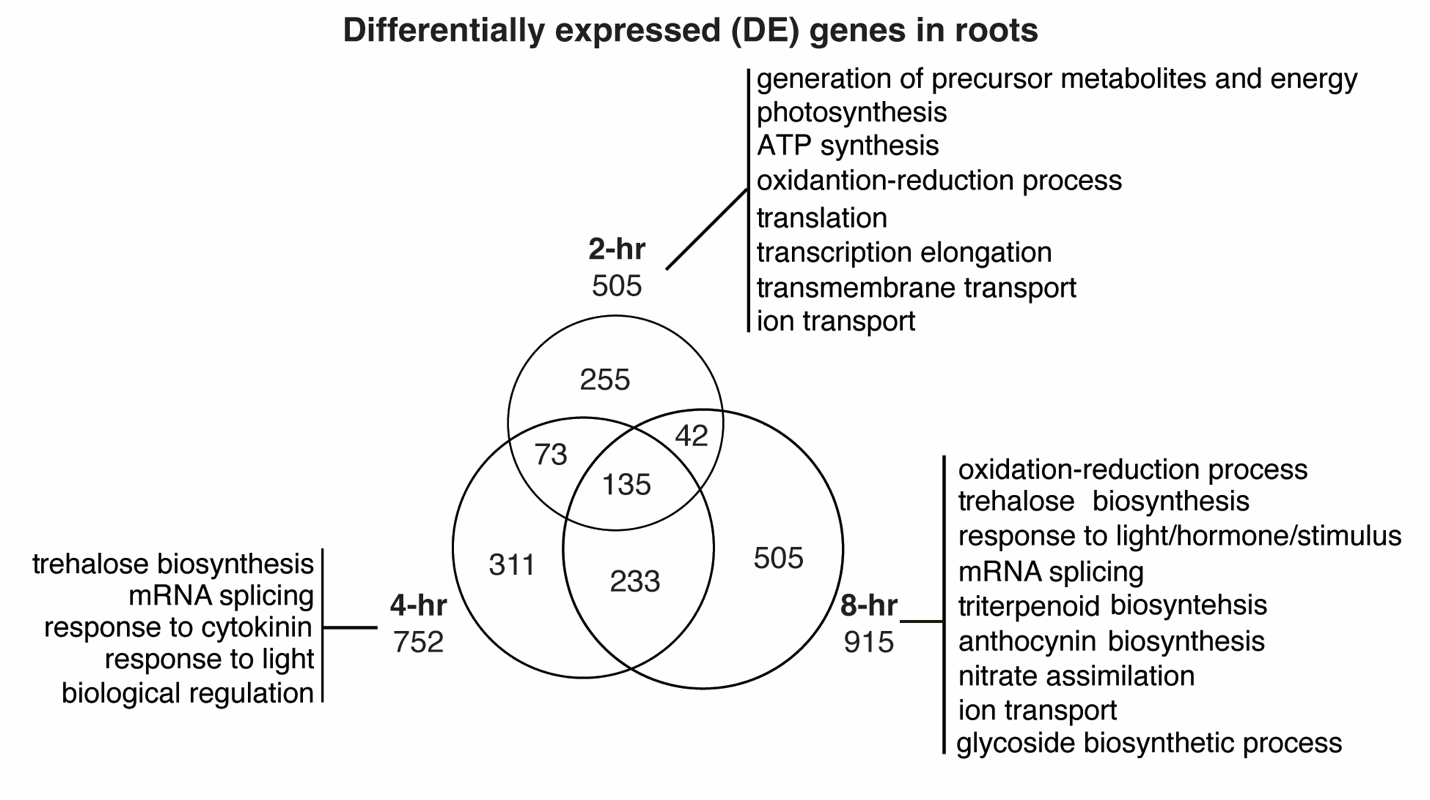


**Supplemental Figure S2.** Venn diagram showing the number and overlap of differentially expressed genes identified in roots at 2, 4 and 8hr after the onset of heterogenous nitrogen treatment in the split-root setup in Arabidopsis. Significantly enriched GO terms are shown next to each gene list.
